# A Comparison of Five-Year Survival Rates Between Thermal Ablation and Hepatic Resection for Colorectal Cancer Metastasis to the Liver: A Systematic Review and Meta-Analysis

**DOI:** 10.14740/wjon2694

**Published:** 2025-12-17

**Authors:** Abdulrahman Alotaibi, Ghala Alshamrani, Hosam Alzobaidi, Ahmad Afandi, Waad Alshamrani, Lana Almuylibi, Wisam Jamal, Waleed Khubzan, Fadi AlBadawi, Noha Guzaiz

**Affiliations:** a Department of Surgery, Faculty of Medicine, University of Jeddah, Jeddah, Saudi Arabia; b Department of Surgery, Faculty of Medicine, Taif University, Taif, Saudi Arabia; c Department of Hepatobiliary Surgery, King Abdullah Medical City, Makkah, Saudi Arabia; d Department of Intervention Radiology, King Abdullah Medical City, Makkah, Saudi Arabia

**Keywords:** Colorectal cancer, Liver metastasis, Thermal ablation, Surgical resection, Five-year survival rate, Meta-analysis

## Abstract

**Background:**

A significant proportion of colorectal liver metastases (CRLMs) are unresectable. This study compares liver resection with thermal ablation.

**Methods:**

The included studies enrolled patients with either resectable or unresectable tumors, depending on tumor characteristics, institutional protocols, and clinical eligibility. All included studies specifically assessed liver metastases from colorectal adenocarcinoma.

**Results:**

The findings of this review found no significant difference in 5-year overall and disease-free survival rates between both approaches (relative risk (RR) = 0.84 (0.54, 1.30), P = 0.35, I^2^ = 94%) and (RR = 1.00 (0.32, 3.13), P = 0.99, I^2^ = 89%), respectively. However, tumor recurrence was higher in the ablation group (odds ratio (OR) = 1.66 (1.06, 2.62), P = 0.03, I^2^ = 10%). Both groups had similar complication rates (OR = 0.34 (0.09, 1.21), P = 0.08, I^2^ = 84%). GRADE certainty was very low for all outcomes. The study quality was heterogeneous, and the Newcastle-Ottawa scale (NOS) score ranged from 5 to 9, indicating a moderate to high risk of methodological quality.

**Conclusion:**

The broad heterogeneity of the quality of studies limits the evidence of thermal ablation. Given these uncertainties, hepatic resection is currently the preferred approach.

## Introduction

Colorectal liver metastases (CRLMs) are one of the most challenging types of cancer for oncologists. The liver is the metastatic site of stage IV colorectal cancer (CRC) in more than half of the patients [1]. Surgery is the accepted therapeutic regimen for CRLM; it has achieved 5-year survival rates of 15-58% [2]. However, not all patients are suitable for this treatment because of factors that may depend more on the characteristics of their cancers, such as tumor bulk, location, and comorbidities, nor is there adequate hepatic functional reserve. Radiofrequency ablation (RFA) and microwave ablation (MWA), guided by imaging techniques, offer a promising approach for patients who may otherwise be ineligible for surgery [3, 4]. Ancillary data from the COLLISION trial indicate that the survival outcomes after thermal ablation may be comparable to those achieved through resecting liver metastases, particularly when the number of metastases is small and confined [5]. In this review, we compared 5-year survival, recurrence rate, and clinical effectiveness between thermal ablation and hepatectomy to better understand the potential role of thermal ablation in treating CRLM.

## Materials and Methods

### Study design

In line with the PRISMA guidelines, we conducted a systematic review and meta-analysis. The study protocol was registered with PROSPERO before initiation (CRD420251006247).

### Outcomes

The primary endpoint of this review was 5-year overall survival in CRLM patients treated with either thermal ablation or hepatic resection. Secondary outcomes consisted of disease-free survival, rate of tumor recurrence, postoperative complications, and length of hospital stay.

### Study selection

We included studies of CRLM patients treated with hepatic resection and/or thermal ablation that reported 5-year survival outcomes. All included studies specifically involved metastases from colorectal adenocarcinoma. Eligible designs included randomized controlled trials (RCTs), cohort studies, propensity-matched analyses, and case-control studies published in English between 2010 and 2024. Only comparative studies that directly compared thermal ablation and hepatic resection were included in the meta-analysis. We excluded studies that reported irrelevant clinical outcomes, relied only on other treatment methods (e.g., chemotherapy) without any surgical ablation intervention, involved primary cancers of other types, were case reports, conference abstracts, review articles with no original data, or articles in languages other than English. The included studies varied in whether they enrolled patients with resectable, unresectable, or mixed CRLM, based on institutional criteria and clinical judgment.

### Search strategy

Two independent authors searched the database, which included PubMed, MEDLINE, EMBASE, Google Scholar, the Cochrane Central Register of Controlled Trials, and ClinicalTrials.gov. The search included studies published between 2010 and 2024. The search algorithm included a combination of Medical Subject Headings (MeSH) and keywords (“colorectal cancer” OR “colorectal neoplasms”) AND (“liver metastasis” OR “hepatic metastasis”) AND (“thermal ablation” OR “radiofrequency ablation” OR “microwave ablation”) AND (“hepatic resection” OR “liver resection”) AND (“Five-year survival” OR “long-term survival”).

Abstracts were assessed for relevance by two independent reviewers using Rayyan, an AI software for systematic reviews [6]. Duplicate records (n = 7) were excluded using Rayyan AI, which automatically detects and flags duplicates for manual verification. Reference lists of relevant studies and reviews were also screened to identify eligible publications. The predefined inclusion and exclusion criteria determined the eligibility of each article included for full-text screening. A third reviewer resolved disagreements about study selection to ensure objectivity and consistency in the selection of studies. We implemented Cohen’s kappa statistic to examine the level of agreement between the two independent reviewers. This measure evaluates inter-rater reliability while adjusting for agreement that occurs by chance, with scores ranging from -1 (complete disagreement) to 1 (perfect agreement). Values above 0.6 typically indicate substantial agreement.

### Data extraction

Two reviewers independently extracted data with a predefined Excel spreadsheet to maintain objectivity and consistency. Data extracted included principal study characteristics (year, author name, type of study, and geographic distribution) and demographic features of study participants (sex distribution, age, and sample size). We also collected data from both the intervention and control groups, including the type of intervention, tumor size, 5-year overall survival rates, local recurrence rates, disease-free survival rates, complication rates, lengths of hospital stays, and durations of follow-up. Any discrepancies in extracted data were resolved by discussion with a third reviewer to ensure accuracy and consistency.

### Quality assessment

We evaluated the methodological quality of the included observational studies using the Risk Of Bias In Non-randomized Studies - of Interventions (ROBINS-I) tool. This tool assesses the risk of bias across seven domains: bias due to confounding, selection criteria, allocation of interventions, deviations from planned interventions, missing data, outcomes, and reported results. We assessed each domain as having a low, moderate, severe, or critical risk of bias, and we assigned an overall risk of bias judgment accordingly [7]. Two reviewers independently assessed each included study, with disagreements being resolved through discussion and consensus. The GRADE assessment tool evaluates the quality of evidence across five key domains (directness of evidence, precision of estimate, consistency of results, risk of bias, and publication bias) [8]. The quality of evidence was rated high or downgraded to moderate, low, or very low. A third reviewer resolved discrepancies. Assessment of publication bias (e.g., funnel plot asymmetry or Egger’s test) was not performed because each meta-analysis included fewer than 10 studies, for which these methods are not recommended due to low statistical power and unreliable interpretation.

### Statistical analysis and data synthesis

We administered the meta-analysis via Review Manager (RevMan) version 5.4 (Nordic Cochrane Centre, Cochrane Collaboration, Copenhagen, Denmark). We included comparative studies (prospective or retrospective) that reported outcomes for both thermal ablation and hepatic resection in patients with CRLM, regardless of the lesion’s anatomical location.

Pooled analyses were performed for each outcome if data were available in at least two studies. We could not use hazard ratios (HRs) for time-to-event outcomes, namely overall survival and disease-free survival, because most included studies reported survival data as the number of patients alive at 5 years. Five-year overall survival was extracted as a fixed time-point outcome because HRs were not reported in most included studies. As individual patient survival data and time-to-event statistics were unavailable, it was not feasible to reconstruct HRs from Kaplan-Meier curves. Therefore, for all binary outcomes, we summarized effects as odds ratios (ORs) with 95% confidence intervals (CIs). Studies judged at critical ROBINS-I risk were excluded from all quantitative syntheses. Sensitivity analyses were conducted by removing studies with serious risk to evaluate their impact on pooled estimates.

We quantified the heterogeneity among studies using the I^2^ statistic and categorized the values as not significant (0-40%), moderate (30-60%), high (50-90%), and significant (75-100%). We used the random-effects inverse-variance model (REML) in all analyses, regardless of the heterogeneity. If meta-analysis was not applicable, we conducted a narrative synthesis to qualitatively summarize the findings.

We performed a random-effects meta-regression analysis using complication rate as a covariate to explore potential sources of heterogeneity. The random-effects inverse-variance model was used to estimate the association between complication rates and 5-year survival outcomes. We apprised the regression coefficients with 95% CIs, P-values, residual heterogeneity statistics (τ^2^ and I^2^), and model R^2^. We also generated a bubble plot to visually illustrate the relationship, with bubble sizes proportional to the inverse variance of each study.

## Results

### Search results

**Figure 1:**
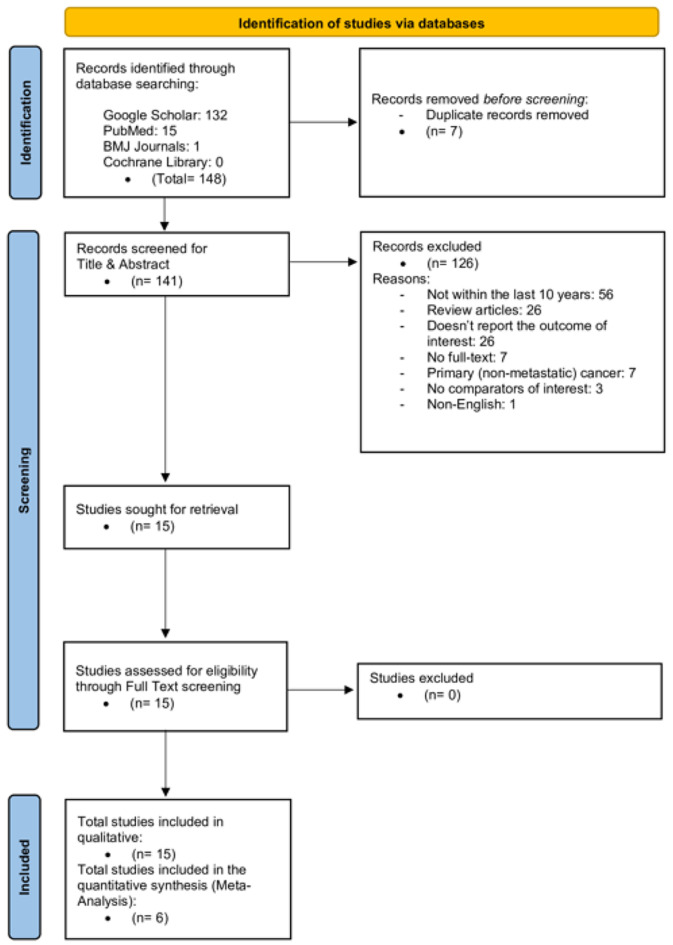
PRISMA chart.

From the database search, we retrieved 148 records: 132 from Google Scholar, 15 from PubMed, one from BMJ Journals, and none from Cochrane Library. We excluded seven duplicates, retaining 141 studies for their title and abstract screening. According to the pre-specified eligibility criteria, we excluded a total of 126 studies: 56 were greater than 10 years old, 26 were review articles, 26 did not report the primary outcome, seven did not have full-text availability, seven included only primary (non-metastatic) CRC, three did not have relevant comparators, and one was published in a language other than English. The inter-rater reliability was substantial, with a Cohen’s kappa coefficient of 0.76, indicating a high level of agreement beyond chance. Following this screening, we included 15 studies (10 retrospective and five prospective) in the qualitative synthesis [9-23], and we included six studies in the meta-analysis [10, 11, 13, 16, 18, 21]. Figure 1 presents a PRISMA diagram of the study selection process.

### Characteristics of included studies and participants

Our 15 studies (Table 1), published between 2014 and 2024, included 10 retrospective studies [9, 10, 14-18, 21-23] and five prospective studies [11-13, 19, 20]. Researchers conducted these studies in Europe (the Netherlands, Germany, Italy, Sweden, and Spain), North America (the United States), and Asia (China, Japan, and South Korea). The summed sample size for all studies was 5,366 patients, with a study population of 36 to 2,008. The average age of the subjects ranged from 52 to 66.8 years, and most studies demonstrated a male predominance. Tumor size was heterogeneous, with mean diameters ranging from 1.4 to 4.5 cm. Trials’ mean follow-up time spanned 16.5 to 90.3 months. Table 2 demonstrates a wide variation in sample sizes across the studies. The number of patients in the ablation groups varied from 52 to 1,005, whereas in the resection groups, it varied from 36 to 1,003. Male-to-female ratios were similar between the two treatment groups. The average age of patients who had an ablation was 58 - 66.9 years, and 55.9 - 66 years for the patients who underwent resection. The length of hospitalization was consistently shorter, ranging from 1 to 9 days in the ablation group, compared with 5 to 8 days in the resection group. The shortest hospital stay for ablation was 1 day [11, 13].

**Table 1:** Study and Participant Characteristics

Author	Year	Design	Location	Sample size	Age (mean)	Males/females	Tumor size (mean, cm)	Follow-up (mean, months)
Vogl et al [9]	2014	Retrospective	Germany	594	61.2	406:188	3.4	22.5
Chiappa et al [10]	2016	Retrospective	Italy	360	59	168:192	2.7	90.3
Dijkstra et al [11]	2021	Prospective	Amsterdam	136	65.1	104:32		29.1
Puijk et al [12]	2022	Prospective	Amsterdam	329	65.3	222:107		16.5
Tinguely et al [13]	2022	Prospective	Sweden	105	66.8	64:41	1.53	48
McEachron et al [14]	2020	Retrospective	USA	36	52	21:15	1.9	28
Hof et al [15]	2016	Retrospective	Netherlands	431	62.9	264:167	3.5	38.6
Huang et al [16]	2021	Retrospective	China	184	57.4	112:72		51.3
Wang et al [17]	2017	Retrospective	China	96	59.5	74:22	4.5	
Masuda et al [18]	2018	Retrospective	USA and Japan	717	58.5	429:288	3	
Knott et al [19]	2021	Prospective	USA	57	60	35:22	1.8	49.3
Valls et al [20]	2014	Prospective	Spain	59	64.1	41:18	2.3	25.3
De Graaff et al [21]	2024	Retrospective	Netherlands	2,008	64.5	1,271:737		76.9
Shady et al [22]	2016	Retrospective	USA	162		92:70		55
Lee et al [23]	2022	Retrospective	South Korea	92	60.2	63:29	1.4	33

**Table 2:** Characteristics of Each Group

Author	Year	Ablation/resection			
		Sample size	Males/females	Age (mean)	Hospital length stay (mean, days)
Chiappa et al [10]	2016	80/280	37:43/131:149	58/60	9/8
Dijkstra et al [11]	2021	100/36	79:21/25:11	66.9/63.3	1/5
Tinguely et al [13]	2022	52/53	33:19/31:22	67.5/66	1/7
Huang et al [16]	2021	98/86	56:42/56:30	58.8/55.9	3.5/7
Masuda et al [18]	2018	116/601	74:42/355:245	58.5/58.4	
De Graaff et al [21]	2024	1,005/1,003	644:361/627:376	64/65	

### Quality assessment

We employed the ROBINS-I tool, as shown in Supplementary Material 1 (wjon.elmerpub.com), to evaluate the risk of bias in the included studies. Overall, we found considerable variation in the methodological quality across the 15 studies. We did not rate any study as having a low risk of bias across all domains.

Confounding (D1) was the most common source of bias, with nearly all studies being judged as either severe or critical due to non-randomized treatment allocation and the influence of clinical or technical factors on intervention choice. Selection bias (D2) was also repeatedly observed, reflecting the retrospective study design and treatment allocation based on surgical candidacy, patient comorbidities, or decisions made by the multidisciplinary team.

We assessed all studies as having a low risk of bias for the classification of interventions (D3), as they clearly defined and consistently applied the treatment modalities (resection vs. ablation). Similarly, deviations from intended interventions (D4) and measurement of outcomes (D6) were generally judged to be of low risk, given that interventions were delivered as planned and outcomes (e.g., overall survival, local recurrence) were measured using objective imaging or clinical follow-up.

Missing data (D5) and selective reporting (D7) were more variable. We rated several studies as having a moderate risk of bias because they reported attrition incompletely, did not register a protocol, or selectively emphasized specific outcomes.

When considering the overall risk of bias, we judged three studies [9, 19, 20] to be at critical risk of bias, primarily because they were single-arm cohorts that lacked a surgical comparator, making them unsuitable for direct comparative synthesis. We deemed seven studies [10, 14, 16-18, 22, 23] to be at serious risk of bias, primarily due to confounding by indication and retrospective treatment allocation. We considered the remaining five studies [11-13, 15, 21] to be at moderate risk of bias, reflecting stronger methodological features such as prospective design, multicenter inclusion, or use of propensity score matching, although residual confounding remained unavoidable. The quality of evidence assessment is shown in Supplementary Material 2 (wjon.elmerpub.com).

### Five-year overall survival

**Figure 2:**
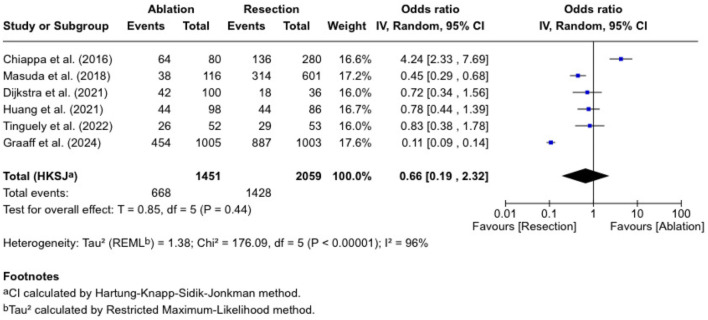
Five-year overall survival.

We performed a meta-analysis of overall survival across six trials as demonstrated in Figure 2. The pooled OR was 0.66 (0.19 - 2.32), with no statistically significant difference (P = 0.44) and high heterogeneity (I^2^ = 96%). Chiappa et al [10] reported an OR of 4.24 (2.33 - 7.69) in favor of thermal ablation, whereas Masuda et al [18] found a lower survival rate in the hepatic resection group (OR = 0.45 (0.29 - 0.68)). In Dijkstra et al [11], Huang et al [16], and Tinguely et al [13], we found ORs close to 1.0, suggesting that the added survival in the two arms is similar. De Graaff et al [21] reported an RR of 0.51 (0.48 - 0.55) in favor of the hepatic resection group, although this paper contributed the largest sample size in our analysis.

While analyzing survival as a binary endpoint does not capture the timing of events, it allowed for consistent pooling across studies with limited time-to-event data.

### Tumor recurrence

**Figure 3:**
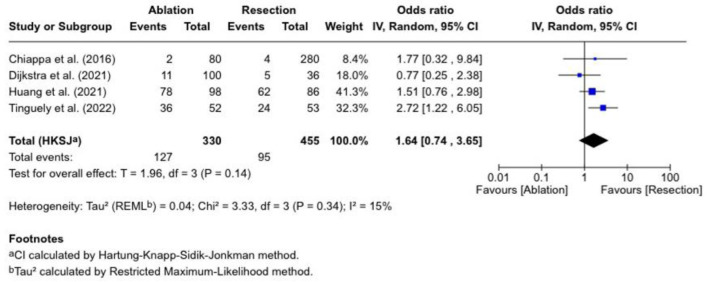
Tumor recurrence.

We evaluated tumor recurrence in four studies, as shown in Figure 3. The pooled OR was 1.64 (0.74 - 3.65), which was statistically insignificant (P = 0.14), and there was low heterogeneity (I^2^ = 15%). Tinguely et al [13] reported an OR of 2.72 (1.22-6.05), indicating a more significant recurrence following ablation. Huang et al [16] reported an OR of 1.51 (0.76 - 2.98). Dijkstra et al [11] reported an OR of 0.77 (0.25 - 2.38), while Chiappa et al [10] had an OR of 1.77 (0.32 - 9.84) (with a wide CI).

### Disease-free survival

**Figure 4:**
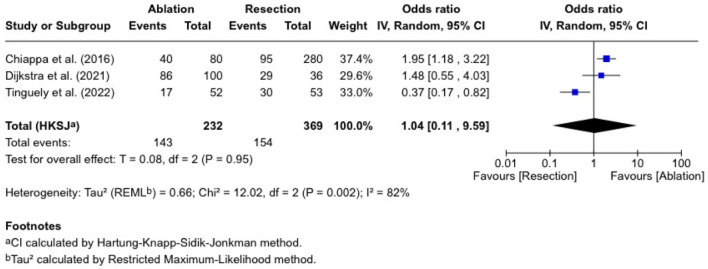
Disease-free survival.

Three studies reported disease-free survival, involving 232 patients who received ablation and 369 who underwent resection (Fig. 4). The combined OR was 1.04 (0.11 - 9.59), which was not significantly different (P = 0.96). Chiappa et al [10] reported an RR of 1.47 (1.12 - 1.94), and Dijkstra et al [11] reported an RR of 1.07 (0.89 - 1.28). In contrast, Tinguely et al [13] reported an RR of 0.58 (0.37 - 0.91). The trials were heterogeneous (I^2^ = 89%; P = 0.002).

### Complication rate

**Figure 5:**
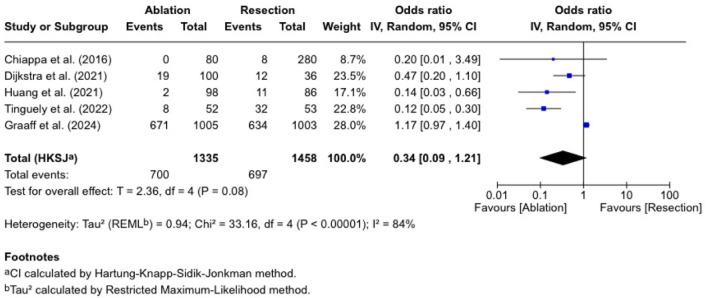
Complication rate.

Five studies evaluated complication rates, with 1,335 patients undergoing ablation and 1,458 undergoing resections (Fig. 5). The OR was 0.34 (0.09 - 1.21), which was statistically insignificant (P = 0.08). The published ORs are varied. Tinguely et al [13] reported an OR of 0.12 (0.05 - 0.30), and Huang et al [16] reported 0.14 (0.03 - 0.66). Dijkstra et al [12] found an OR of 0.47 (0.20 - 1.10), Chiappa et al [10] reported an OR of 0.20 (0.01 - 3.49), and de Graaff et al [21] reported an OR of 1.17 (0.97 - 1.40). There was considerable heterogeneity (I^2^ = 84%, P < 0.00001).

**Figure 6:**
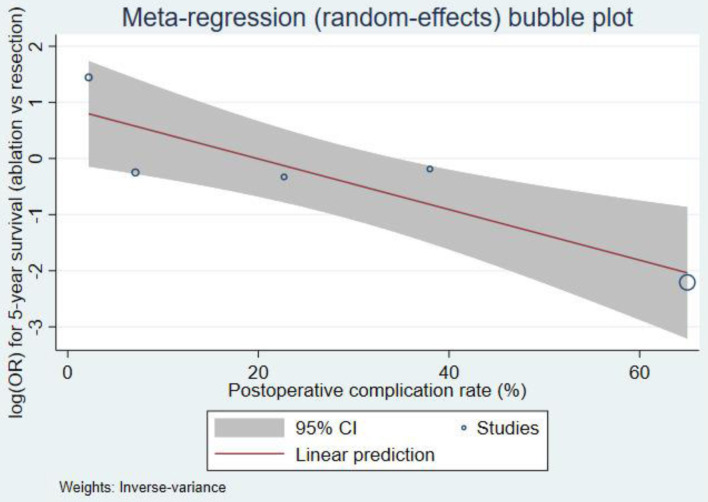
Meta-regression bubble plot.

The meta-regression found a significant negative association between complication rate and 5-year survival (coefficient = -0.045, 95% CI: -0.072 to -0.018, P = 0.001). The model explained a substantial proportion of heterogeneity (R^2^ = 74.1%), with residual heterogeneity remaining considerable (τ^2^ = 0.434; I^2^ = 79.9%). The bubble plot (Fig. 6) illustrates this inverse relationship, showing that studies with higher complication rates tend to report lower 5-year survival rates.

## Discussion

This systematic review and meta-analysis primarily compared the long-term outcomes of thermal ablation with hepatic resection for CRC metastases, with a specific focus on 5-year overall survival, tumor recurrence, disease-free survival, and complications. We included 15 studies in our qualitative synthesis and six in our meta-analysis, totaling 5,366 patients.

Table 3 demonstrates the summary of our pooled analysis. There is no statistically significant difference in 5-year survival between thermal ablation and hepatic resection (RR = 0.84 (0.54 - 1.30)). The heterogeneity was significantly high, primarily due to variations in patient selection, tumor burden, and ablation techniques. Notably, Chiappa et al [10] demonstrated an advantage for ablation, while de Graaff et al [21] demonstrated superior survival with hepatic resection. Despite other review articles that consider hepatic resection the gold standard for CRLM [24-26], they recommend using ablation as an adjunctive therapy in frail patients or for those with unresectable tumors.

**Table 3:** Summary of Pooled Analysis

Outcomes	Effect size	CI	Heterogeneity I^2^	P value
Overall survival	RR: 0.84	0.54 - 1.30	94%	0.35
Tumor recurrence	OR: 1.66	1.06 - 2.62	10%	0.03
Disease-free survival	RR: 1.00	0.32 - 3.13	89%	0.99
Complications	OR: 0.34	0.09 - 1.21	84%	0.08

CI: confidence interval; OR: odds ratio; RR: relative risk.

Tumor recurrence was significantly higher in the ablation group (OR = 1.66 (1.06 - 2.62), P = 0.03, I^2^ = 10%), supporting findings from van Amerongen et al [24], which found that ablation, despite its minimally invasive nature, has a higher likelihood of incomplete tumor eradication and residual microscopic disease. Our results further confirm those from Yang et al [25], which indicated that thermal ablation, particularly RFA, carries a higher local recurrence risk than resection, likely due to insufficient margin clearance.

Our pooled analysis for disease-free survival indicated no significant difference (RR = 1.00 (0.32 - 0.3.13)). The substantial heterogeneity, however, suggests that patients’ selection criteria likely contributed to this outcome. Our findings align with those of Shady et al (2019) [22], which suggest that the success rate of ablation is significantly influenced by tumor size and proximity to prominent vasculature, thereby affecting long-term disease control.

Our pooled analysis revealed no statistically significant difference in complication rates (OR = 0.34 (0.09 - 1.21)). However, Tinguely et al [13] and Huang et al [16] had lower complication rates for the ablation group. Others have concluded that hepatic resection had more postoperative morbidity [24-27].

The COLLISION trial [5], a rigorously designed phase 3 RCT, provided high-quality evidence suggesting the potential non-inferiority of thermal ablation compared to hepatic resection for small-sized (≤ 3 cm), technically resectable CLM. Among 296 patients randomized, the overall survival rates were nearly identical between ablation and resection (HR, 1.05; 95% CI, 0.69 - 1.58; P = 0.83). Notably, the ablation group had a safer profile, with fewer adverse effects compared to the resection group (19% vs. 46%, P < 0.0001), and the rates of serious complications were significantly lower with ablation (7% vs. 20%).

Crucially, the COLLISION trial stratified and balanced patients across established prognostic factors, including tumor size, number of metastases, baseline carcinoembryonic antigen (CEA), synchronous versus metachronous presentation, nodal status of the primary tumor, and receipt of neoadjuvant chemotherapy. This methodological rigor enabled meaningful subgroup analysis and ensured high internal validity. In contrast, most of the retrospective observational studies included in our meta-analysis (e.g., [10, 11, 16, 21]) lacked consistent reporting or adjustment for these critical variables. Notably, retrospective studies, such as those by Chiappa et al [10] and de Graaff et al [21], used different criteria for selecting ablation or surgery, which affected the comparability of outcomes. As a result, we were unable to contextualize survival and recurrence in relation to tumor biology or clinical risk profile. This limitation likely contributed to the heterogeneity observed across studies. It may explain why our pooled analysis indicated higher recurrence rates with ablation, while in selected patients, COLLISION suggested equivalent oncologic efficacy in a well-selected, balanced cohort.

Furthermore, unlike the COLLISION trial, which employed strict procedural protocols and follow-up regimens, many included studies did not standardize the ablation modality (e.g., RFA vs. MWA) [16, 19] or control for tumor characteristics such as proximity to vasculature or tumor size, factors known to influence recurrence [22, 23].

Additionally, the high heterogeneity in our meta-analysis, particularly for 5-year survival and disease-free survival, is attributable to wide clinical and methodological variation across studies. Important differences included tumor burden, mean lesion size, bilobar disease distribution, the use of neoadjuvant or adjuvant chemotherapy, and differences in follow-up duration (ranging from 16.5 to 90.3 months). Although we conducted a meta-regression using complication rate as a covariate, the small number of available studies (n ≤ 6) limited the statistical power to evaluate additional variables. For this reason, we supplemented the quantitative assessment with narrative stratification of key contributors to heterogeneity. While the COLLISION trial offers strong robustness, our meta-analysis reflects routine clinical practice, including patients often excluded from RCTs. This broader perspective provides valuable context to inform treatment decisions across a more diverse range of patient populations.

The inclusion of studies at serious ROBINS-I risk, although necessary due to the limited availability of high-quality comparative evidence, may introduce residual confounding. This risk is reflected in the very low GRADE certainty ratings across outcomes. However, the consistency of sensitivity analyses suggests that these studies did not unduly influence the pooled estimates.

Given the substantial heterogeneity and the very low certainty of evidence (GRADE), the observed similarity in survival outcomes should be interpreted cautiously. The outcomes of thermal ablation are strongly influenced by patient selection, tumor size, lesion location, and operator expertise, emphasizing that these factors are key in determining survival rates.

Importantly, the overall interpretation of our findings must be viewed through the lens of the ROBINS-I and GRADE assessments. Most included studies were judged to have a serious or critical risk of bias, primarily due to confounding by indication, retrospective treatment allocation, and incomplete reporting of prognostic variables. These methodological limitations directly undermine the comparability of treatment groups and likely contribute to the substantial inconsistency observed in survival and recurrence outcomes. Consistent with these concerns, the GRADE assessment rated the certainty of evidence for all outcomes as very low, driven by serious risks of bias, high heterogeneity, imprecision in effect estimates, and indirectness in reporting. Therefore, although the pooled estimates suggest no statistically significant difference in 5-year survival between ablation and resection, this finding should be interpreted with extreme caution. The available evidence is insufficient to establish equivalence or non-inferiority, and true treatment effects may differ substantially from the pooled results.

### Limitations

We acknowledge several limitations. Firstly, the evident heterogeneity observed in our meta-analysis, particularly for the survival and disease-free survival endpoints, is a marker of variability in study designs, patient populations, and treatment regimens. Secondly, 10 out of 15 included studies being retrospective may result in selection bias since the resected patients would have been healthier or possessed improved tumor biology. In addition, several of the prospective studies had relatively small sample sizes (ranging from 57 to 329 patients), which limits the statistical power and generalizability of their findings. Furthermore, some studies did not uniformly describe discrepancies in ablation modality (RFA versus MWA), and most did not distinguish between them when reporting recurrence outcomes, thereby preventing any meaningful comparison between the two techniques. Additionally, follow-up periods varied randomly between trials, ranging from 16.5 to 90.3 months, which may have influenced recurrence and survival rates.

A limitation of the review is that we were unable to formally assess publication bias. Funnel plots and statistical tests such as Egger’s regression require a minimum of 10 studies to yield interpretable results; all outcomes in this meta-analysis included six or fewer studies. Therefore, publication bias could not be reliably evaluated. This limitation has been reflected in the GRADE assessment. Another important limitation is the use of ORs for 5-year survival. While this approach allowed harmonization of the available data, it does not capture the time-dependent nature of survival or differences in censoring between studies. HRs would provide a more accurate reflection of comparative survival, but these were not available in the included literature and could not be reliably reconstructed. Consequently, the pooled 5-year survival findings should be interpreted cautiously.

As mentioned earlier, we found that most studies lacked detailed reporting of key prognostic factors of CRLM. They inconsistently reported variables such as tumor size, number of lesions, serum CEA levels, synchronous versus metachronous presentation, nodal status of the primary tumor, and receipt of neoadjuvant chemotherapy. The lack of these factors constrained our capacity to conduct rigorous stratified analyses and meaningful comparisons with the COLLISION trial. Furthermore, because the included studies did not report HRs, we were unable to perform a time-to-event survival meta-analysis using HR, which would have been the ideal approach.

Lastly, the inability to confirm whether all patients included in the eligible studies were candidates for both hepatic resection and thermal ablation. Most studies did not report exclusion criteria or specific contraindications for either treatment, which contributed to selection bias. As a result, differences in outcomes, such as survival and recurrence, may reflect underlying patient selection rather than the treatment effect alone. This limitation should be taken into account when interpreting the comparative findings of this review. Future studies should aim to standardize eligibility criteria and report them transparently.

### Conclusion

This systematic review and meta-analysis show that although hepatic resection remains the benchmark treatment for CRLM, thermal ablation may provide comparable survival for carefully selected patients, but with a higher risk of recurrence. However, due to the high heterogeneity and low certainty of evidence, this apparent comparability should be viewed with caution and not interpreted as procedural equivalence. The choice of optimal treatment should depend on patient factors, tumor characteristics, and the capabilities of the treating institution. Treatment needs to be patient-specific. Future research should establish clear patient selection criteria and continue to improve ablation technology to achieve better long-term outcomes.

## Supplementary Material

Suppl 1 Risk of bias assessment.

Suppl 2 Quality of evidence assessment.

## Data Availability

All data were obtained from previously published studies; no new data were generated in this review.
