# Supplementary figures and images for: A Comparison of Five-Year Survival Rates Between Thermal Ablation and Hepatic Resection for Colorectal Cancer Metastasis to the Liver: A Systematic Review and Meta-Analysis
Source: World J Oncol. 2025 Dec 17;17(1):95–105. doi: 10.14740/wjon2694 (PMC12758054; doi:10.14740/wjon2694)

**Suppl 1.** Risk of bias assessment.

**
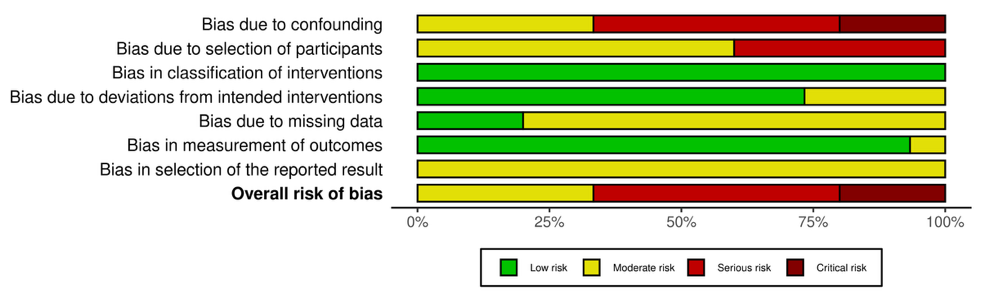

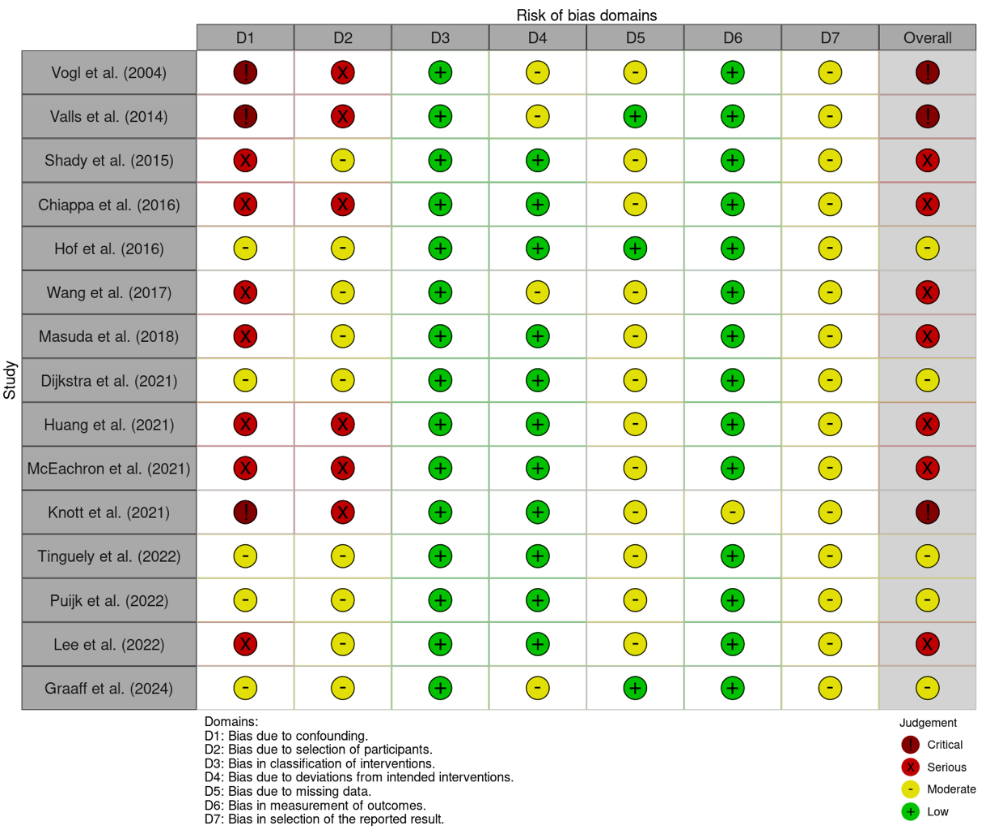
**

Supplement: Suppl 1 — Risk of bias assessment. [file wjon-17-01-095-s001.docx]
