# Supplementary material for: A Comparison of Five-Year Survival Rates Between Thermal Ablation and Hepatic Resection for Colorectal Cancer Metastasis to the Liver: A Systematic Review and Meta-Analysis
Source: World J Oncol. 2025 Dec 17;17(1):95–105. doi: 10.14740/wjon2694 (PMC12758054; doi:10.14740/wjon2694)
Supplement: Suppl 2 — Quality of evidence assessment. [file wjon-17-01-095-s002.docx]

**Suppl 2.** Quality of evidence assessment.

| **Table summarizing the GRADE assessments and highlighting the confidence in the estimates of effect.** | | | | | | | | |
| --- | --- | --- | --- | --- | --- | --- | --- | --- |
| **Outcomes** | **Quality assessment** | | | | | **Summary of Findings** | | |
|  |  |  |  |  |  | **Effect** | **Overall** | **Certainty** |
|  | **Risk of Bias^** | **Consistency *** | **Precision §** | **Directness ¥** | **Publication bias** |  |  |  |
| **5 Years Overall Survival** | No serious limitations | Serious limitations | Some limitations | No serious limitations | Not assessable | OR= 0.84 (95% CI, 0.54, 1.30) | No difference | Very low |
| **Tumor Reoccurrence** | No serious limitations | No serious limitations | No serious limitations | No serious limitations | Not assessable | OR= 1.66 (95% CI, 1.06, 2.62) | Significant difference | Very low |
| **Disease-free Survival** | No serious limitations | Serious limitations | Serious limitations | No serious limitations | Not assessable | OR= 1.00 (95% CI, 0.32, 3.13) | No difference | Very low |
| **Complication Rate** | No serious limitations | Serious limitations | Some limitations | No serious limitations | Not assessable | OR= 0.34 (95% CI, 0.09, 1.21) | No difference | Very low |
| **^ Risk of bias was downgraded if the high-risk domain was likely to affect the outcome. * Consistency was downgraded if the heterogeneity was statistically significant [p< 0.05]. § Precision was downgraded if the confidence interval crossed the zero, indicating no difference. ¥ Directness was downgraded if different measures were used. OR: Odds ratio. CI: Confidence interval.** | | | | | | | | |
